# Design of Semi-Rigid Composite Highway Pavements Using Cementitious Grouting and Porous Asphalt

**DOI:** 10.3390/ma18194636

**Published:** 2025-10-09

**Authors:** Sevil Kofteci, Mansor Nazary, Ahmad Khaled Masbah, Halil Ibrahim Burgan

**Affiliations:** Department of Civil Engineering, Akdeniz University, Antalya 07070, Turkey; skofteci@akdeniz.edu.tr (S.K.); mansor870.nazary@gmail.com (M.N.); khalidmesbah2015@gmail.com (A.K.M.)

**Keywords:** cementitious grout, composite highway, fluidity, porous asphalt, semi rigid pavement

## Abstract

Due to the increasing volume of traffic on the world’s highways, researchers have been searching for new composite techniques and methods to develop durable and cost-effective pavement structures. The semi-rigid pavement is a composite pavement consisting of a porous asphalt mix with air voids between 25 and 30% and a high-fluidity cementitious grout. In this study, different cementitious grout mixes were prepared. Then a porous asphalt mix with almost 30% air void content was designed. After evaluating the workability, mechanical strength, and volume stability of the prepared grout mixes, the most suitable mix is proposed to fill the voids in the porous asphalt mix. Finally, the prepared semi-rigid pavement specimens were subjected to various tests to evaluate the performance characteristics of the designed pavement. The research concludes that the grout mixture ratio proposed in this study has good grouting ability and the semi-rigid pavement has superior performance characteristics.

## 1. Introduction

Flexible and rigid pavements are the most common types of pavement used throughout the world. The difference between these two types of pavement is based on the nature of the material that makes up the upper layers. Flexible pavement is a combination of aggregate and bitumen and is generally known for its good ride quality and high recoverable deformation. Rigid pavement is a mixture of cement and aggregates known for its longer life and high mechanical strength. Increasing traffic flows and traffic loads mean that these types of pavement cannot meet the requirements of motorways in the long term. Researchers have therefore been looking for new techniques and methods to develop durable and cost-effective pavement structures. The Semi-Rigid Pavement (SRP) is a composite pavement consisting of a porous asphalt mix and cementitious grout that can combine the advantages of rigid and flexible pavements.

SRP was first developed in France in 1960 [1]. The development of this pavement was carried out by the company Jean Lefebvre Enterprises; they proposed it to replace the rigid and flexible pavement [2]. Between 1970 and 1980, interest in this type of pavement developed in all European countries, Africa, and North America [3,4,5]. Investigations by Steyawan [6] and Mayer [7] showed that SRP has high durability, capacity, and freeze/thaw resistance. Hong investigated the freeze–thaw durability of semi-flexible pavement mixtures. He used two different grouting materials (early-strength (ES) and high-strength (HS)) in porous asphalt, the results indicating that enhanced freeze–thaw resistance of an SFP mix can be achieved by incorporating a high-strength grouting material into a porous asphalt [8]. Di Mascio found in his “Economic evaluation of cement grouted bituminous mixes for air-port Pavements” research that the economic opportunity of cement-grouted bituminous mix solutions strongly depends on traffic loads. He compared the construction and maintenance costs of a cement grouted bituminous mix pavement and a traditional flexible pavement at two different airports (a taxiway and a helipad one). The cost of the helipad surface paved with cement-grouted bituminous mix is higher than that obtained for the traditional pavement [9]. Mechanical properties, durability properties, and microstructural characterization of semi-flexible pavement were assessed by Raza. He concluded that the incorporation of cementitious grout in semi-flexible pavements enhances their moisture resistance and overall durability. Furthermore, the utilization of grout materials—such as high-strength and early-strength formulations combined with polymer binders—strengthens the resistance of semi-flexible pavements to freeze–thaw cycles and moisture-induced damage [10]. Ding et al. [11] found that the high- and low-temperature properties of SRP materials were superior to those of conventional asphalt pavements. Ling et al. concluded that SRP had anti-skid and anti-fatigue properties and better resistance to low-temperature cracking [12]. Fang et al. used a polycarboxylate-based superplasticizer in the SRP grout and reported an improvement in performance as well as a balance between flexibility and stiffness properties [13]. Bang et al. evaluated the effects of flowability and compressive strength of cementitious grout on SRP; test results showed that the flowability property of cementitious grout was the most important factor affecting the mechanical properties of SRP [14]. Lundstrom et al. compared the performance of flexible, rigid, and SRP and reported that SRP has better performance characteristics than flexible and rigid pavements. In general, SRP consists of an open-graded asphalt layer with 25 to 35% air voids, which is filled with cementitious grouting materials. This mix allows the pavement to be used for heavy traffic roads, industrial areas, and airports. SRP is constructed in two stages. In the first stage, the porous asphalt is designed with an air void content of 25 to 35%, using the same method as for the design of a normal porous asphalt pavement. In the second stage, grout is designed to fill the air void content of the porous asphalt. The good performance of SRP depends on the air void content of the porous asphalt and the fluidity of the grouting material. A grout with insufficient fluidity will not fully penetrate the air void content of the porous asphalt and may cause the pavement to crack. Therefore, the air void content of the porous asphalt and the fluidity of the grouting materials are two important factors that should be considered during the SRP design process.

The aim of this laboratory Investigation Is to design a porous asphalt layer for semi-rigid pavement and to develop a cementitious grout and evaluate its contribution to the resulting semi-rigid pavement. Therefore, it can be said that this experimental study consists of three parts. In the first phase of this research, different cementitious grout mixes were prepared with sand, cement, two types of superplasticizers, and two different water-cement ratios. Four laboratory tests, including a flow test, compressive strength test, flexural strength test, and drying shrinkage test, were carried out to evaluate the workability, mechanical properties, and volume stability of the cementitious grouts. In the second phase, porous asphalt with almost 30% air void content was developed. The air void content of the porous asphalt was calculated according to the two different additives. In addition, the optimum bitumen content of the porous asphalt was calculated using an empirical equation based on aggregate gradations. In the third phase, the performance of the semi-rigid pavement was investigated by applying the most suitable cementitious grout to 65 mm porous asphalt specimens.

## 2. Experimental Program

### 2.1. Cementitious Grout

#### 2.1.1. Materials

CEM I 42.5 R cement from AS Cimento Ltd. Antalya, Turkey was used in the research program. Table 1 shows the chemical and physical properties of the cement used in the experimental study. The specific gravity and fineness of the cement are 3.08 g/cm^3^ and 3357 cm^2^/g, respectively, and the chemical composition of the cement is given in the table. Natural limestone < 0.6 mm was used as an aggregate in the cementitious grout. Table 2 shows the grading limits of the aggregate used in this study.

Two types of polycarboxylate-based high-range water-reducing superplasticizers, named X and Y, were used in this study. These superplasticizers had the same polymer structure but different dry matter contents. Some properties of the superplasticizers obtained from their manufacturers are given in Table 3.

#### 2.1.2. Preparation Cementitious Grout

A total of 12 different cementitious grout mix compositions were prepared using an automatic mixer with a mixing time of 5 min. The mixes were prepared with two different water/binder ratios (0.65 and 0.70) and two types of superplasticizers in three dosages (0.25%, 0.5%, and 1% of cement). The codes and mix compositions of the cementitious grouts are given in Table 4.

#### 2.1.3. Workability of the Grout

**Figure 1:**
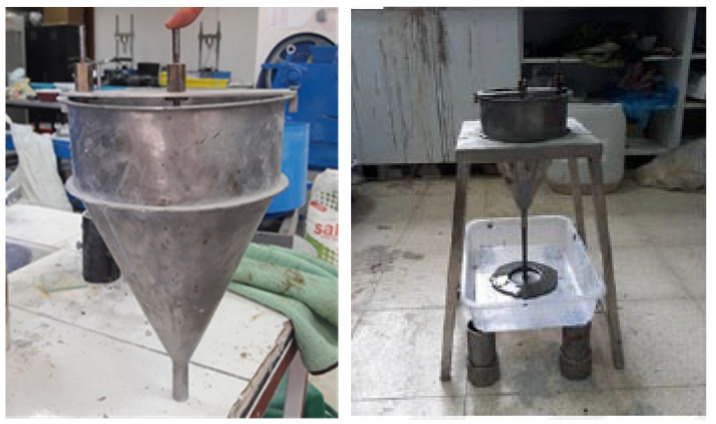
Flow cone for hydraulic cement grout flow test.

The fluidity of the cementitious grout mixtures was measured by the flow cone test. The fluidity of grouts is commonly measured by flow time, and the test is based on ASTM C939 [15]. The internal diameter of the V-funnel used in this study is 12.7 mm (Figure 1). The time taken for 1725 mL of cementitious grout to pass through the V-funnel was defined as the flow time in this investigation.

#### 2.1.4. Mechanical Properties of the Grout

**Figure 2:**
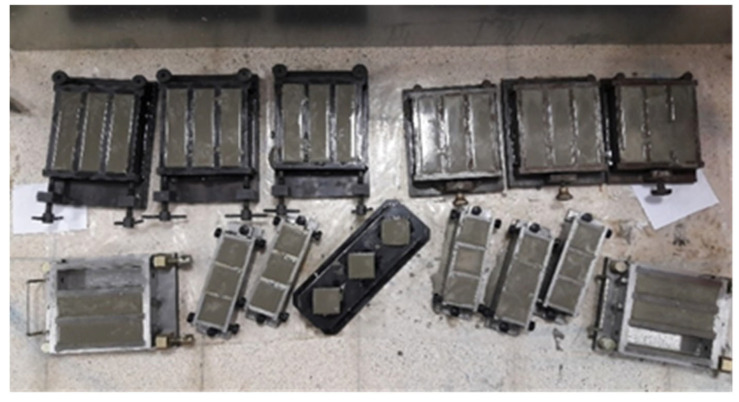
Cementitious grout specimens prepared for the tests.

To determine the mechanical performance of the cementitious grouts, compressive and flexural strength tests were carried out using a universal testing machine with a capacity of 2500 kN. For compressive strength, 50 mm cube specimens of each mix were prepared and tested at 3, 7, and 28 days in accordance with ASTM C109 [16]. For the flexural strength of cementitious grouts, 40 × 40 × 160 mm prism specimens were prepared for each mix design and tested at 3, 7, and 28 days in accordance with ASTMC348-18 [17]. Figure 2 shows the specimens prepared for the grout performance tests.

#### 2.1.5. Volume Stability of The Grout

**Figure 3:**
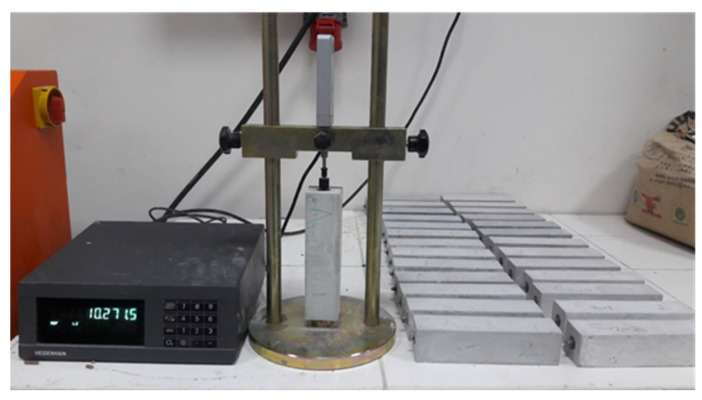
Shrinkage test apparatus.

The volume stability of the cementitious grout was determined by a drying shrinkage test. The test was performed according to ASTM 596-09 [18]. The drying shrinkage values were measured at the ages of 2, 4, 11, 18, and 25 days of the specimens. Figure 3 shows the experimental set-up for the shrinkage test.

### 2.2. Porous Asphalt

#### 2.2.1. Materials

In this investigation, the aggregates used in the design of porous asphalt, which provides SRP’ skeleton, are crushed limestone. The physical properties of aggregates were evaluated by the conducting of various tests as shown in Table 5.

In this study, we used bitumen 50/70 in the design of the porous asphalt because of its high viscosity. Normally, low penetration-grade bitumen is more practical in hot areas because it may be more resistant to the high temperatures. On the other hand, it may slightly affect the air void content of the porous asphalt. As can be seen from Table 6, the results of the tests carried out on bitumen 50/70 meet all the specification limits defined in the Turkish Highway Construction Specifications.

#### 2.2.2. Preparation Porous Asphalt

**Figure 4:**
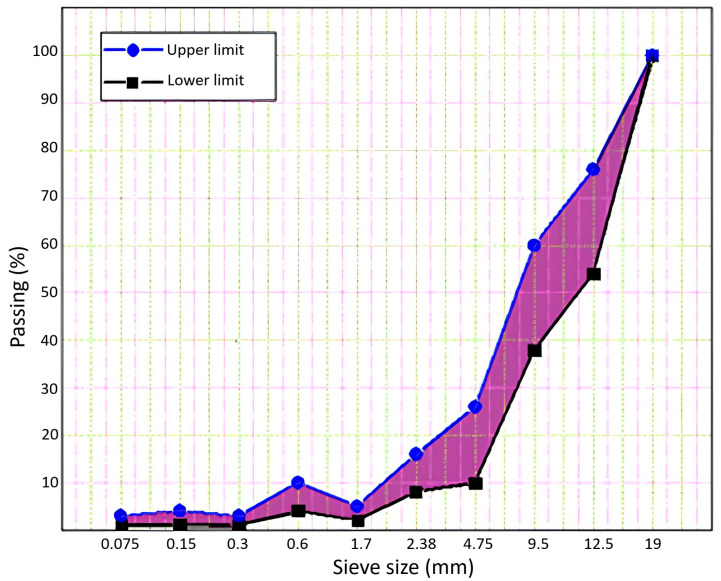
Reference aggregate gradation limits.

In this experimental study, we used reference aggregate gradation limits according to the semi-rigid pavement project carried out in the United States of America to calculate the mixing formula for porous asphalt aggregates [2]. The reference aggregate gradation limits are shown in Figure 4.

According to the specified aggregate limits, the blending formula for porous asphalt aggregates, which indicates the percentage of each aggregate size, was calculated by the method determined in THCS, as shown in Table 7.

The modified additive was blended completely with bitumen at temperatures above 115 °C (1 to 4% by weight of bitumen) to reduce the mixing and compaction temperature of asphalt mixture without the reduction in pavement quality. The reduction in the mixture’s compaction temperature may allow it to travel longer haul distances. In this research, bitumen was modified with a modified additive before adding to the mixture (3% by weight of bitumen) at a temperature 135 °C.

Fiber is an additive that is used to stop binder drainage between aggregates during transportation and storage. In this investigation, we used cellulosic fiber (0.3% by weight of mixture) with the dry method to evaluate its effect on the air voids content of porous asphalt. Table 8 shows the physical properties of cellulosic fiber.

#### 2.2.3. Determination Optimum Bitumen Content (OBC)

Optimum bitumen content shows the percentage of bitumen that balances the mixing properties for each aggregate gradation and bitumen type. In this study, the optimum bitumen content of porous asphalt was calculated by the empirical Equation (1) [2], which is based on the properties of each aggregate size calculated in the optimum blend formula.

(1) $$OBC=3.25\left(\alpha \right)\Sigma^{0.2}$$

where

α=2.65/Gs, Gs total specific gravity of aggregates in the mixture;

Σ= Specific surface area =0.21G+5.4S+7.2s+135f;

G= Percentage of aggregate retained on sieve 4.75 mm;

S= Percentage of aggregates passing through sieve 4.75 mm and retained on sieve 0.6 mm;

B= Percentage of aggregates passing through sieve 0.6 mm and retained on sieve 0.075 mm;

f= Percentage of aggregates passing through sieve 0.075 mm.

(2) $$Gs=\frac{100}{\frac{P_{1}}{G_{1}}+\frac{P_{2}}{G_{2}}+\ldots \frac{P_{n}}{G_{n}}}$$

where $P_{1},P_{2},\ldots ,P_{n}$ are the percentage of aggregates used in the mixture and $G_{1},G_{2},\ldots ,G_{n}$ are apparent specific gravity of aggregates.

#### 2.2.4. Design of Porous Asphalt with Marshall Specimens

**Figure 5:**
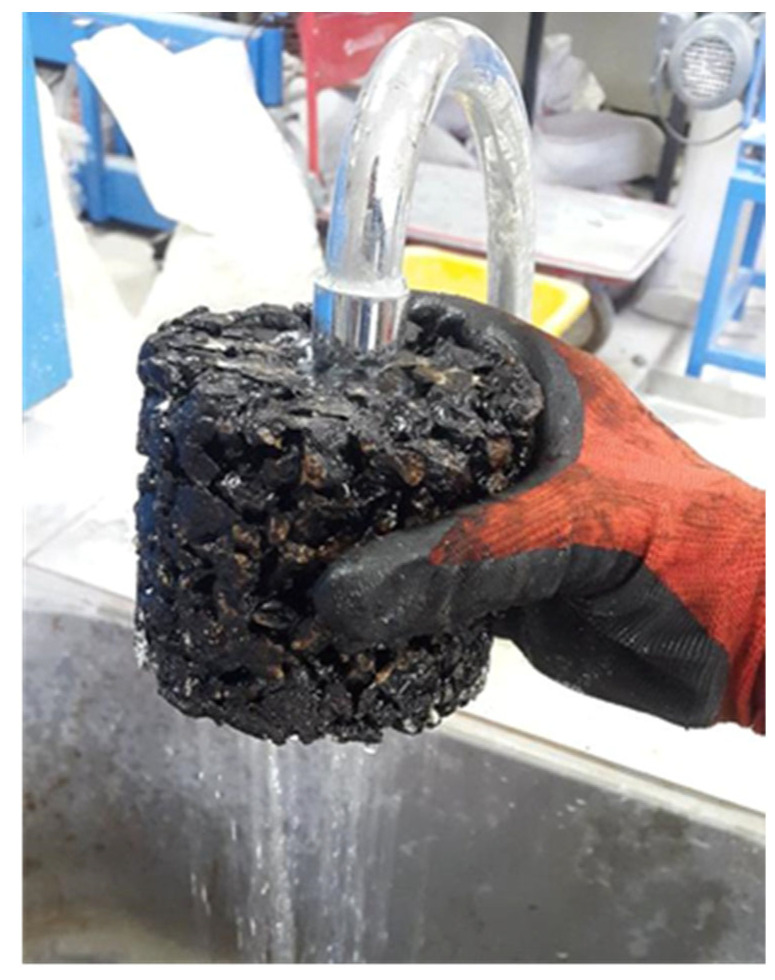
Porous asphalt prepared as Marshall specimen.

Porous asphalt mixture was prepared by use of OBC and percentage of each aggregate size, which was calculated in the optimum blend formula. Immediately after mixing, the asphalt mixture was placed in a Marshall mold and compacted on one side with 25 blows [2] from a 4.5 kg Marshall hammer at a temperature between 110 and 120 °C in order to obtain more air voids content in Marshall specimens. Figure 5 shows the Marshall specimen, which has more air voids.

After the preparation of Marshall specimens, air voids content (VTM) for each compacted specimen was calculated by the following equations.

(3) $$VMA=100-100\frac{W_{t}}{\left(\frac{\pi }{4}D^{2}H\right)\left(G_{mm}\right)}$$

where

$W_{t}$ is the dry weight of the specimen, D is the diameter of the specimen, H is the height of the specimen, and $G_{mm}$ is the maximum theoretical specific gravity.

(4) $$G_{mm}=\frac{P_{mm}}{\frac{P_{s}}{G_{s}}+\frac{P_{b}}{G_{b}}}$$

where

$P_{mm}$ is the total weight of the mixture (in percent), $P_{s}$ is the percentage of aggregates by weight of the total mixture, $P_{b}$ is the percentage of bitumen by weight of the total mixture, $G_{s}$ is the total apparent specific gravity of aggregates used in the mixture, and $G_{b}$ is the specific gravity of bitumen.

### 2.3. Tests of Semi-Rigid Pavement

**Figure 6:**
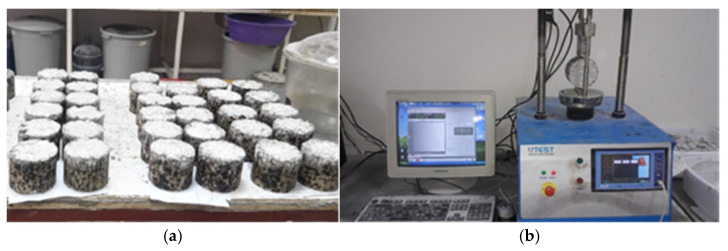
SRP specimens and indirect tensile test apparatus, (**a**) SRP specimens; (**b**) Indirect tensile test apparatus.

In this phase of the study, the proposed mixture design proportions of cementitious grout and porous asphalt were used. Porous specimens of 101.6 mm in diameter and 80 mm in height were prepared as Marshall specimen which have more air voids than traditional Marshall specimens. The bottom of the molds was covered to prevent leakage of the grout. When the temperature of the specimens dropped below 30 °C, the prepared cementitious grout was spilled on the specimen. Then, grout freely filled the voids of the specimen by the gravity action. Then the designed grout penetrated the air voids of porous asphalt under the influence of gravitational force and its natural fluidity, without the application of any external pressure, and the percentage of grout penetration almost exceeded 95%, as determined by comparing the specimen weight before and after grouting (Figure 6a).

Various laboratory tests, including Marshall stability and flow, indirect tensile strength, compressive strength, Cantabro, as well as freeze–thaw resistance tests, were conducted to evaluate the performance of the SRP. It is worth mentioning that three specimens were tested for each curing age category (3, 7, and 28 days), and the values reported in the discussion section for each category represent the average rates derived from these replicates for all the tests, including Marshall stability and flow, indirect tensile strength, compressive strength, Cantabro, as well as freeze–thaw resistance tests.

The Marshall method of the mixture was conducted to determine the stability and flow of the SRP specimens according to ASTM D6927 [32]. For this test, SRP samples were prepared and allowed to be air-cured for 3, 7, and 28 days. In order to break specimens, they were immersed in a water tank at 60 °C for 40 min and then tested in a Marshall Testing Machine (Figure 6b).

The tensile strength of SRP was determined by using an indirect tensile strength testing approach in this study. For this test, cylindrical specimens were allowed to be air-cured for 3, 7, and 28 days. The test was performed by placing the specimens horizontally between two plates and applying a vertical load of 50 mm per minute across its diameter until it broke. The ultimate load was recorded, and Equation (5) was used to calculate the tensile strength.

(5) $$T_{s}=\frac{2000\times P}{\pi \times t\times D}$$

To determine the compressive strength property of SRP, specimens were subjected to compressive strength tests according to ASTM C39/C39M-18 after 3, 7, and 28 days of air curing. A thin layer of high-strength gypsum plaster was used to achieve the planeness of the cylindrical specimens.

Mass losses of SRP specimens were calculated using the Cantabro test. Specimens were exposed to air curing for 3, 7, and 28 days. After the air-curing process was completed, the weights of the specimens were measured. Then they were placed into the Los Angeles drum machine, and the machine was rotated 300 gyrations with a speed of 30 rpm. The damaged specimen was removed from the drum machine and measured again, and the percentage of weight loss was calculated.

To estimate the freeze–thaw resistance of SRP, three specimens were subjected to three cycles and three specimens to five cycles of freeze and thaw. Each cycle consists of a total of 1440 min, 900 min of freezing at −12 °C, and 540 min of thawing at +28 °C. Specimens were subjected to indirect tensile strength after completing the cycles.

## 3. Results and Discussions

### 3.1. Test Results, Analysis and Discussion of The Cementitious Grout

#### 3.1.1. Workability of The Grout

**Figure 7:**
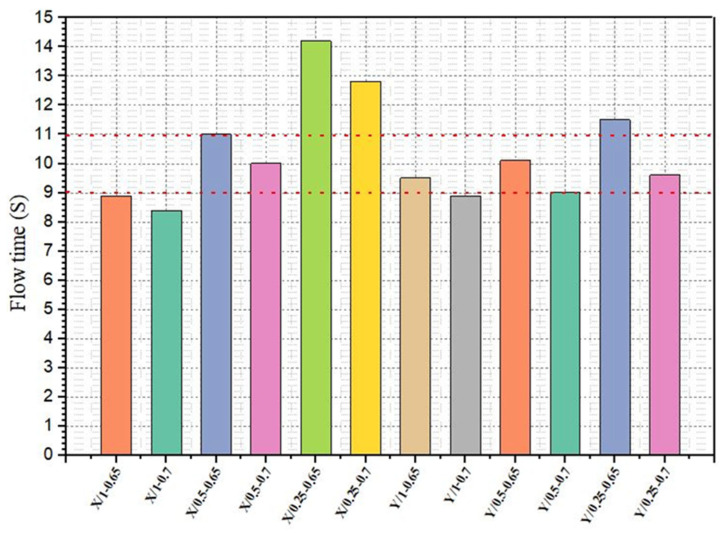
Fluidity test results of cementitious grout mixtures.

The cementitious grout should have sufficient fluidity to fill the pores of the porous asphalt mixture. According to the literature research, the flow time of grout used in SRP should be between 9 and 11 s without applying any compression and vibration [33,34,35,36]. The fluidity test results are shown in Figure 7. As would be expected, irrespective of the type of superplasticizer, the flow time of grouts decreased with increasing the dosage of superplasticizer. The mixture X/1-0.7 has the lowest flow time compared to other mixtures. However, this mixture has seen excessive segregation. Bang et al. have proposed the flow time of grout used in SRP as 12 s [14]. According to the results obtained in this context, the type of superplasticizer and W/C ratio play important roles in grout preparation. The selection of a high-quality water-reducing additive is very important for the successful application of cementitious grout. The interaction between superplasticizer dosage and W/C ratio indicates that higher superplasticizer dosages, even with a higher W/C ratio, can maintain relatively low flow times, as seen in Y/1.0-0.7, demonstrating the superplasticizer’s effectiveness in improving fluidity despite increased water content. In contrast, mixtures with a lower superplasticizer dosage and higher W/C ratio, such as X/0.25-0.7, demonstrate longer flow times, indicating that the combination of less superplasticizer and more water reduces fluidity.

#### 3.1.2. Mechanical Performance of The Grout

**Figure 8:**
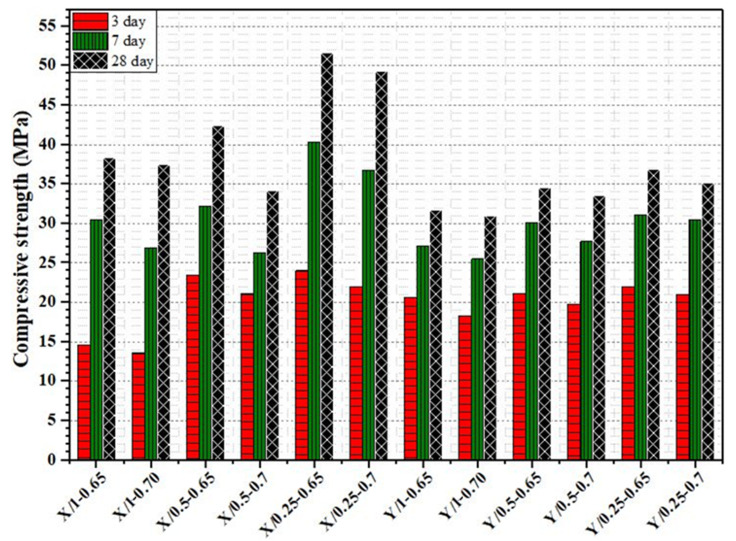
Compressive strength test results cementitious grout mixtures.

The test results of the compressive strength of all cementitious grout mixtures are presented in Figure 8. According to results, the increase in superplasticizer dosage and W/C ratio decreases the compressive strength of grout. The 3-day strengths of all mixtures are all above 12 MPa. The 28-day compressive strength of grout mixtures prepared using Y superplasticizer additive ranges from 35.1 MPa to 49.3 MPa, while the 28-day compressive strength of grout mixtures prepared using Y superplasticizer additive ranges from 38.3 MPa to 51.6 MPa. Grout mixtures prepared with superplasticizer X show higher strength than those prepared with superplasticizer Y at all dosage levels. The 28-day compressive strength of the X-superplasticizer mixtures is consistently greater. The maximum 28-day compressive strength of the X-superplasticizer mixture is about 15 MPa higher than that of the Y-superplasticizer mixture.

**Figure 9:**
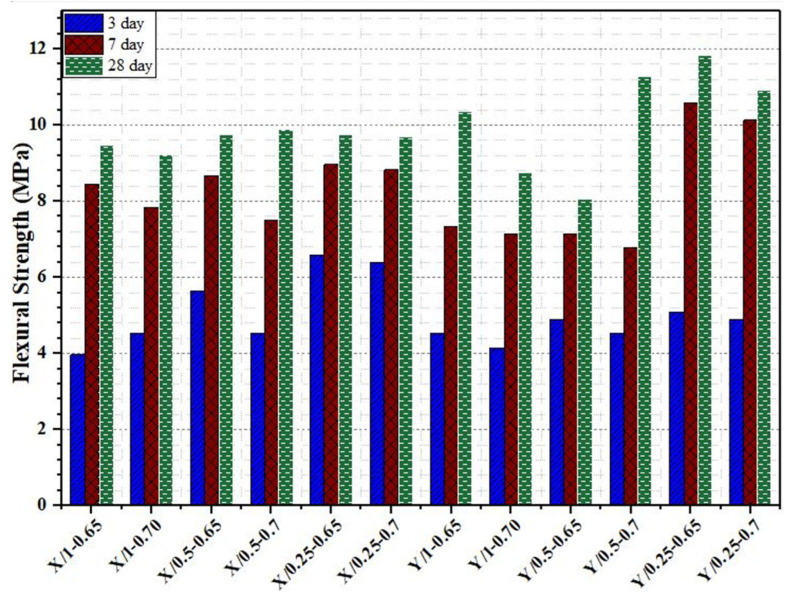
Flexural strength test results of cementitious grout mixtures.

The flexural strength development of the cementitious grout mixtures at 3, 7, and 28 days is presented in Figure 9. In general, all grout mixtures exhibited a continuous increase in flexural strength with curing age, indicating the progressive hydration of cement and the positive contribution of the superplasticizers to strength development. Among the tested mixtures, Y/0.25-0.65 achieved the highest 28-day flexural strength (12 MPa), while Y/0.5-0.65 recorded the lowest values (8 MPa). This indicates the strong effect of both the type of superplasticizer and its dosage on the flexural performance of the grout. When comparing the two superplasticizers, mixtures prepared with Y-superplasticizer generally demonstrated better flexural strength compared to those with X-superplasticizer. For instance, Y/0.25-0.65 and Y/0.25-0.7 exceeded the performance of the X-mixtures with the same dosages, suggesting that the Y-superplasticizer enhanced the tensile-related behavior of the grout more effectively. However, the dosage effect was also evident: while lower dosages of Y (0.25) led to higher flexural strength, higher dosages (0.5) reduced strength. Overall, the results confirm that the type and dosage of superplasticizer significantly affect the flexural performance of cementitious grout.

#### 3.1.3. Volume Stability of The Grout

**Figure 10:**
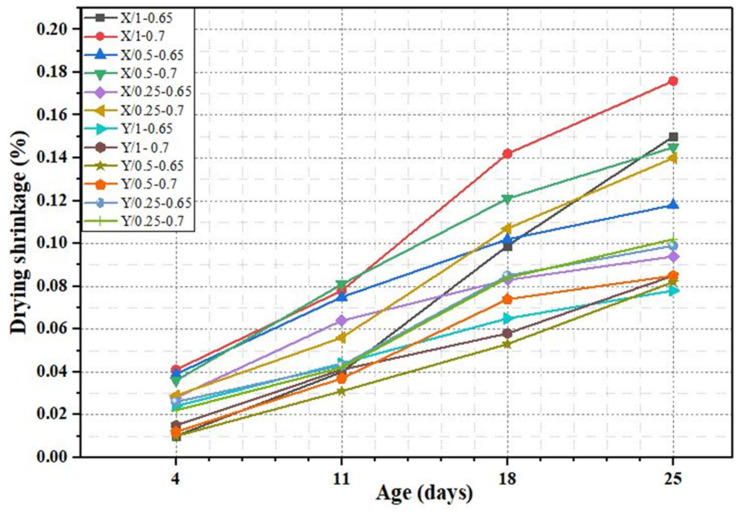
Drying shrinkage test results of cementitious grout mixtures.

Drying shrinkage of cementitious grout mixtures, as shown in Figure 10, was influenced by the W/C ratio of mix proportion and dosage content of superplasticizers. A higher W/C ratio and superplasticizer dosage led to a high value of drying shrinkage. From the results obtained, grout mixtures prepared with X superplasticizer, when the W/C ratio changed from 0.65 to 0.7 at the same dosage of additive, the drying shrinkage value enlarged from 0.15% to 0.17% after 25 days, while when the superplasticizer dosage changed from 0.25% to 1% at the same W/C ratio, the drying shrinkage value enlarged from 0.14% to 0.17%. In the grout mixtures prepared with Y superplasticizer, when the W/C ratio changed from 0.65 to 0.7 at the same dosage of superplasticizer, the drying shrinkage value enlarged from 0.078% to 0.085% after 25 days, but the change in dosage content of superplasticizer does not affect it significantly. By comparing the types of superplasticizer, it was seen that the grout mixtures prepared with Y superplasticizer have lower drying shrinkage values.

### 3.2. Test Results, Analysis, and Discussion of Porous Asphalt

**Figure 11:**
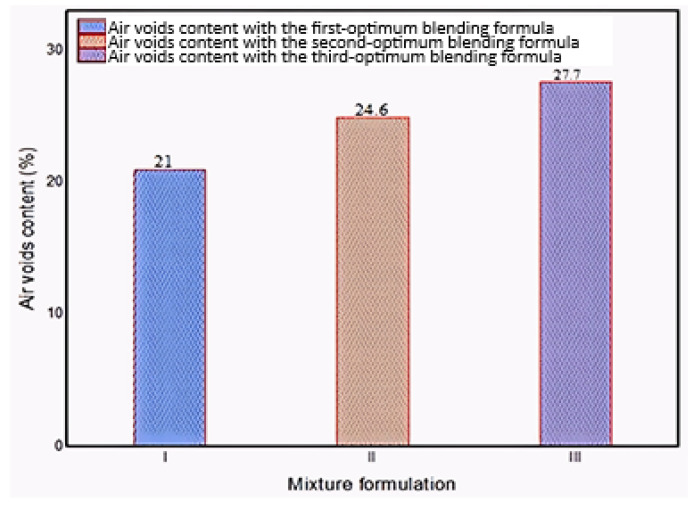
Air voids content of Marshall specimens in different mixtures.

**Figure 12:**
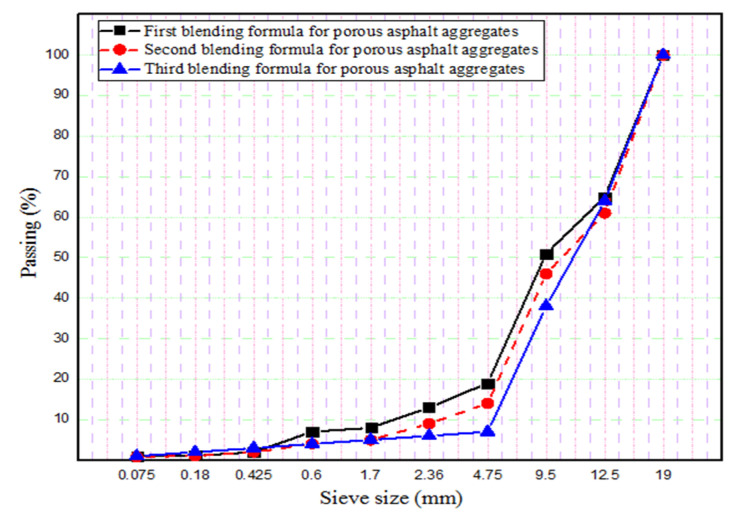
Aggregate gradations used in three different asphalt mixtures.

**Figure 13:**
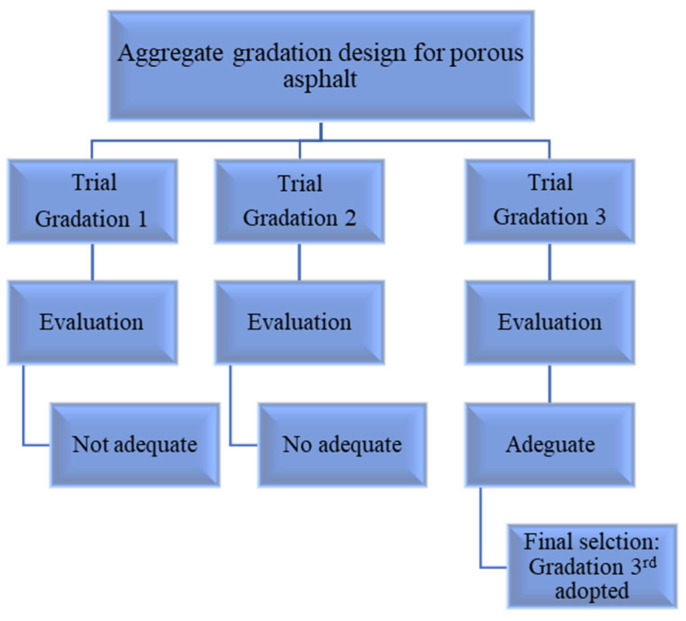
Flowchart of aggregate gradation design for porous asphalt.

In the first stage of this investigation, a porous asphalt mixture was prepared according to the OBC, and the percentage of each aggregate size was calculated in the first optimum blending formula. As shown in Figure 11, the Marshall specimens, prepared with the first blending formula do not provide the intended percentage air voids content of porous asphalt (25–35%). Therefore, second and third aggregate gradations were defined in order to obtain more air voids content in the Marshall specimens as shown in Figure 12. OBC for each blending formula was separately calculated according to the percentage of each aggregate size (3.88%, 3.69%, and 3.65%). The porous asphalt mixture prepared with the third aggregate gradation provided the required air voids content of the asphalt skeleton, which can be used in the SRP. So, the third blending formula for porous asphalt aggregates was chosen as an optimum aggregate gradation after the evaluation of the first and second blending formulas (Figure 13). As inferred from the OBC values and three different aggregate gradations, OBC decreased in three different asphalt mixtures, respectively, because of the low percentage of fine aggregates. The more specific surface area of fine aggregate may be the main reason for the decreasing values of OBC.

**Figure 14:**
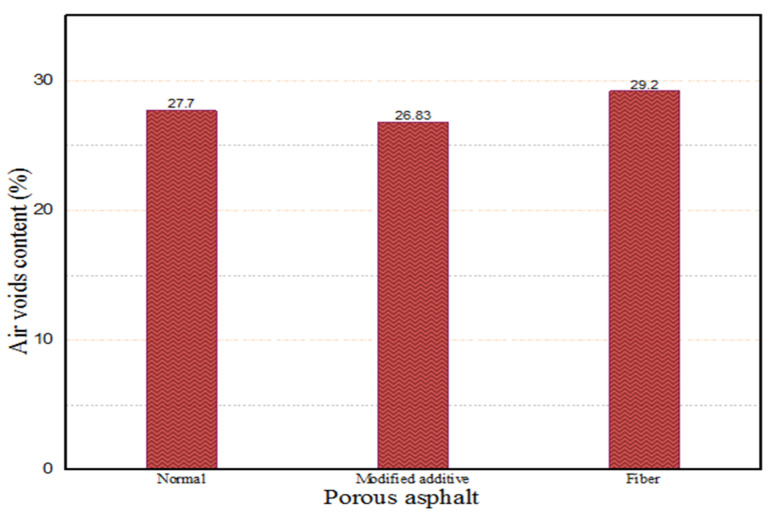
Air voids content of porous asphalt with different additives.

In addition, the air voids content of the porous asphalt mixture was evaluated by the use of two different additives (fiber 0.3% by weight of the mixture and modified additive 3% by weight of bitumen) after the determining of the optimum blending formula. As shown in Figure 14, Marshall specimens prepared with fiber are more porous than modified additive specimens and specimens without additive. Since the fibers are not uniformly spread and clump together, they hinder proper aggregate packing and compaction, which leads to increased voids in asphalt. On the other hand, the incorporation of cellulose fibers may result in increased air void content within asphalt mixtures. This effect is primarily attributed to the fibers’ absorption of asphalt binder, which reduced the effective binder available for aggregate coating and subsequent compaction. Conversely, modified additives in asphalt mixtures reduce binder stiffness and improve the coating of aggregate particles, thereby enhancing compaction efficiency and leading to a reduction in air void content. This was an expected result because the application of fiber in the mixture increases the air voids content of the asphalt mixture [37]. At the same time, the existence of fiber in the asphalt mixture increases the mixture permeability coefficient [38]. Therefore, it may result in more air voids content of the asphalt mixture. On the other hand, the Marshall specimens designed with modified additives provided less air void content due to the reduction in mixture compaction strength. The viscosity of bitumen with modified additive increases at low temperatures (less than 90 °C) and decreases at high temperatures (more than 90 °C) [39]. Therefore, the low viscosity of bitumen may lead to a decrease in mixture compaction strength. As a result of various experimental tests, the third aggregate gradation, OBC (3.65%), and fiber (0.3% by weight of mixture) were selected as the optimum factors that can provide almost 30% air voids content of porous asphalt.

### 3.3. Performance of the Semi-Rigid Pavement

**Figure 15:**
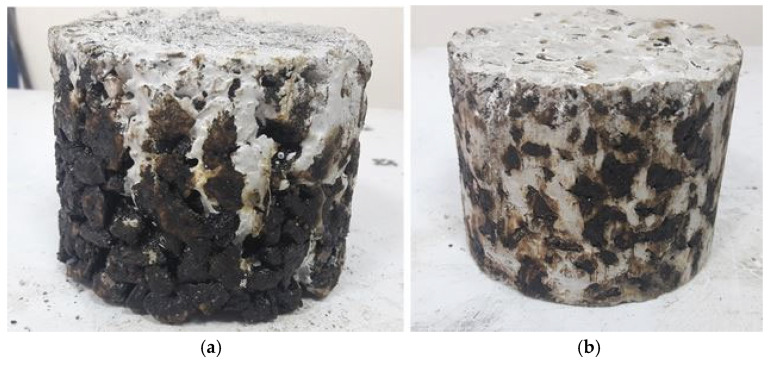
Grouting ability of SRP specimens (**a**) specimen filled with grout mixture X/0.25-0.7 (**b**) specimen filled with grout mixture Y/0.5-0.7.

The selection of an appropriate cementitious grout for semi-rigid pavement (SRP) requires balancing three key properties: fluidity, volume stability, and strength. According to the literature, the fluidity of cementitious grout and the air content of porous asphalt play the most critical role in SRP design, as the grout must penetrate the air voids of the porous asphalt under gravitational force and fill at least 95% of the voids [2,40]. In this study, the initial grout mixture X/0.25-0.7, with a flow time of 12.8 s, failed to penetrate the porous asphalt (Figure 15a), while the Y/0.5-0.7 mixture successfully filled the voids completely (Figure 15b), confirming its superior workability. Y/0.5-0.7 also achieved a flow time within the recommended 9–12 s range (Figure 7), ensuring full infiltration without the segregation observed in highly fluid mixes like X/1-0.7. Furthermore, it showed much lower drying shrinkage (≈0.085%) than comparable X-based mixtures (≈0.17%) (Figure 10), providing better dimensional stability and reducing the risk of cracking or debonding. While Y-based mixtures generally had slightly lower 28-day compressive strength (35–49 MPa) than X-based ones, Y/0.5-0.7 still offers sufficient strength (Figure 8) along with acceptable flexural performance (Figure 9) to contribute to the load-bearing capacity of SRP. Overall, Y/0.5-0.7 provides the best balance among fluidity, stability, and strength and is therefore selected as the optimum grout mixture for SRP ap-plications.

**Figure 16:**
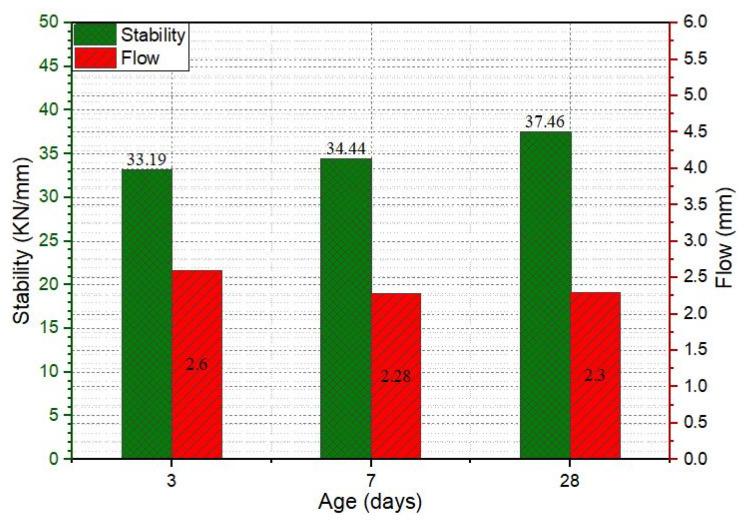
The Marshall stability and flow of SRP specimens.

Stability value refers to the structural strength of the pavement. Prepared SRP specimens were subjected to the Marshall stability test after 3, 7, and 28-day air curing, and the results are shown in Figure 16. As indicated in Figure 16, the stability value of specimens increased by increasing curing time and reached 37.46 kN until 28 days. The integration of cementitious grout within the porous asphalt results in a composite structure that effectively brings together the mechanical advantages of both cementitious grout and porous asphalt. Cementitious grout, with its high compressive strength, enables the composite to withstand heavy traffic-induced stresses. At the same time, the asphalt skeleton contributes flexibility and promotes strong bonding with the aggregate particles, thereby minimizing friability and enhancing the overall durability of the SRP composite. According to Turkish Highway Construction Specification 2013 (THCS), an adequate Marshall stability value for the wearing course of flexible pavement is 900 kg (8.89 kN). The Asphalt Institute [41] proposes a minimum stability value of 8 kN; similarly, ERA [42] indicates a minimum stability value of 9 kN for a very heavy traffic class pavement. Deshmukh obtained the maximum stability value of SRP as 22.7 kN [43]. By comparing the Marshall stability of SRP results of the current study with the literature review, it can be concluded that the mechanical properties of cementitious grout improved the performance of the SRP mixture. Flow value is an indication of the plasticization of the deformation of pavement under traffic load. According to THCS, the flow value for wearing courses is between 2 and 4 mm. As shown in Figure 15, the flow value is inside the limits of THCS.

**Figure 17:**
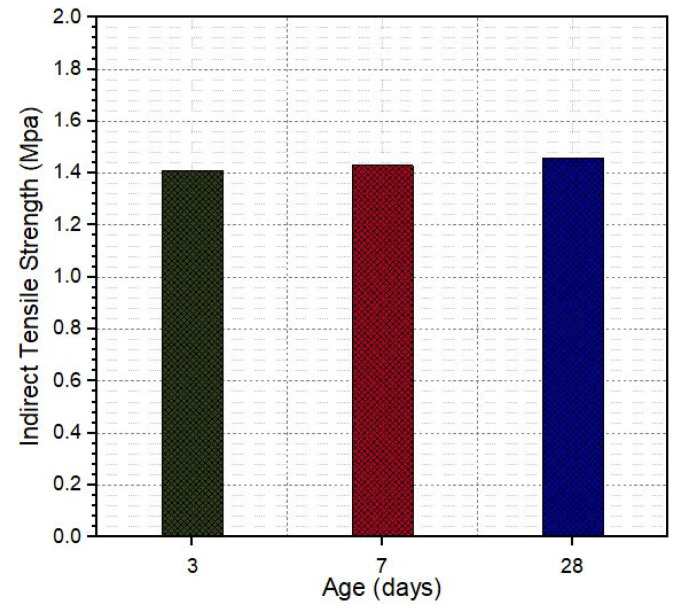
The tensile strength of SRP specimens.

The indirect tensile strength test was performed to determine the fracture strength and stiffness of the pavement mixtures. The test results of SRP specimens at different curing times are shown in Figure 16. As indicated in Figure 17, curing time has no significant effect on the tensile strength; the maximum strength obtained at 28 days is 1.46 MPa. Hulala determined the tensile strength of porous asphalt with a % of air content as 0.18 MPa [44]. The cementitious grout used in the design of SRP specimens in the current study had 10.92 MPa flexural strength at 28 days of curing, so we can conclude the flexural strength of grout increased the indirect tensile strength of SRP (Figure 17).

**Figure 18:**
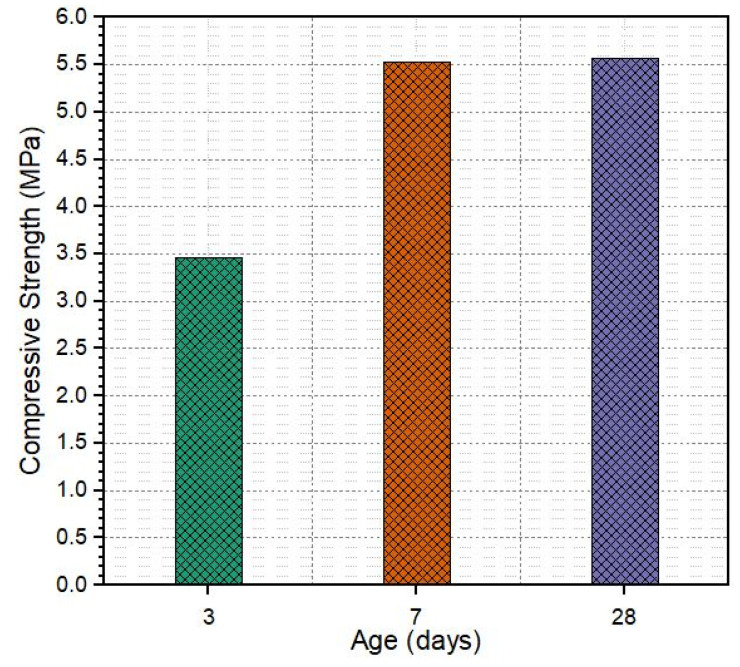
The compressive strength of SRP specimens.

The compressive strength of SRP was calculated by dividing the maximum load obtained by the cross-sectional area of the specimen, and the result is shown in Figure 18. According to the literature review, Densiphalt^®^ [45] and DuraTough^®^ [46], which are commercial products, declared in their user manuals that the maximum compressive strength is 7 MPa and 5 MPa after 28 days of curing for SRP. As seen in Figure 18, the compressive strength of the current study is 5.55 MPa at 28 days of curing. Obtained results fall within this range. Therefore, by comparing with the literature review it can be said that the obtained compressive strength is adequate for SRP.

**Figure 19:**
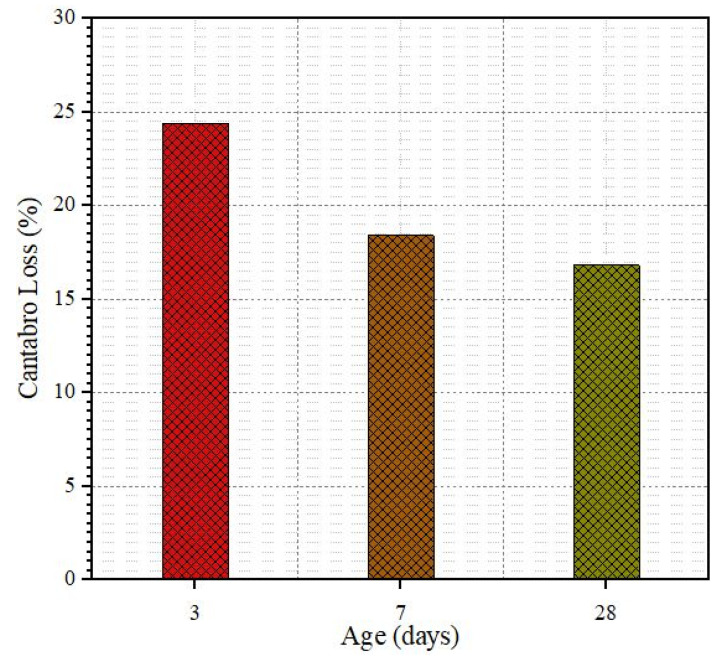
The mass loss of SRP specimens.

The abrasion resistance or Cantabro test was conducted to estimate the mass loss of SRP specimens at three different air-curing times. Figure 19 shows the Cantabro test results for 3, 7, and 28 days. As indicated in the figure, the mass loss of SRP specimens decreased over time due to the grout hardening over this time. As the resistance of grout material increases over time, it may lead to better bonding between grout material and porous asphalt mixture.

**Figure 20:**
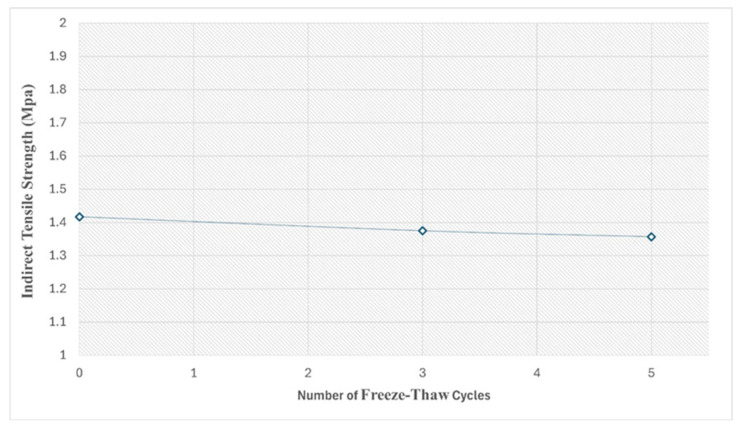
The freeze–thaw test results of SRP specimens.

One of the major problems for pavements is the cold climate, which causes early deterioration of the pavement layer. The freeze–thaw test was conducted to evaluate the moisture damage resistance of SRP. Specimens were subjected to indirect tensile strength after 3 and 5 cycles of freeze–thawing, and the results are shown in Figure 20. Based on the test results, it is seen that the strength losses after 3 and 5 freezing and thawing cycles are 2.1% and 3.2%, respectively. The incorporation of cementitious grout within the porous asphalt enhances the material’s resistance to freeze–thaw cycles by minimizing moisture-induced damage. The grout infiltrates and fills the interconnected voids of the asphalt skeleton, thereby reducing permeability and restricting water infiltration, which is the principal factor contributing to frost damage. Furthermore, the high stiffness and compressive strength of the cementitious grout provide structural reinforcement for the SRP composite and withstand the internal stresses provided by the volumetric expansion of freezing water. This means that SRP can be applied in cold climatic regions in terms of Life Cycle Assessment (LCA) [47].

## 4. Conclusions

In this investigation, cementitious grouts and porous asphalt mixtures were first designed under laboratory conditions, and then the air void content of the porous asphalt mixture was filled with the proposed grout mixture. The research outcomes demonstrate that the grout mix with superplasticizer Y at 0.5% dosage and a water/cement ratio of 0.7 provided the appropriate balance between fluidity, volume stability, and mechanical strength. Its best penetration capacity ensured nearly complete filling of the porous asphalt pores, thereby forming a robust composite structure. On the asphalt part, the use of a coarse aggregate gradation comprised of cellulose fiber produced the targeted high air void content (30%), which enabled deep grout penetration and strong mechanical interlock.

By successfully combining these materials, the resulting SRP specimens showed markedly enhanced Marshall stability, compressive strength, and freeze–thaw resistance compared with conventional flexible pavements. This confirms that the synergistic combination of a rigid grout and a flexible asphalt skeleton can overcome the traditional trade-off between strength and flexibility in pavement systems. Notably, the mechanical performance of the developed SRP matched or exceeded values reported for commercial products such as Densiphalt^®^ and DuraTough^®^, while also exhibiting improved durability under moisture and low-temperature conditions. This highlights the practical potential of the proposed mix design as a cost-effective alternative for heavy-traffic roads and cold climate regions.

Yet, this study was limited to laboratory conditions and short-term performance testing. Long-term behavior, including fatigue, cracking, and rutting resistance should be confirmed by field trials under actual traffic loads and varying environmental conditions. The research should also investigate using alternative cementitious materials or green binders in the future to enhance SRP construction sustainability further.

In summary, this research helps in the evolution of SRP technology by verifying a grout–asphalt mixture that achieves optimal structural performance and laying the ground for additional full-scale application and optimization studies.

## Figures and Tables

**Table 1:** Chemical and physical properties of cement.

Chemical Properties		Physical Properties	
SiO_2_ %	18.85	Specific gravity (g/cm^3^)	3.08
Al_2_O_3_ %	9.94	Blaine specific surface (cm^2^/g)	3357
Fe_2_O_3_ %	3.42	Initial setting time	195
CaO %	62.22	Final setting time	285
MgO %	1.57	Mechanical properties	
SO_3_ %	2.9	Compressive strength	
Na_2_O %	0.18	2 Day (MPa)	22.2
K_2_O %	0.62	7 Day (MPa)	38.4
Cl %	0.0135	28 Day (MPa)	54.3

**Table 2:** Aggregate gradation for cementitious grout.

Sieve Size (mm)	0.6	0.425	0.18	0.075
Percent passing by weight (%)	100	99.6	34.2	1.5

**Table 3:** Properties of polycarboxylate ether-based superplasticizer admixtures.

Properties	X	Y
Appearance	Brown	Brown
pH, 25 °C	4.1	4.5
Density, (g/cm^3^)	1.07	1.1
Dry material content (%)	34.54	28.91
Water soluble chloride (%)	0.02	0.03
Alkali content (%)	Fair	Fair
FT-IR Spectrum/Effective component	Fair	Fair

**Table 4:** Mix proportions of the cementitious grout mixtures (kg/m^3^).

Mixture	Superplasticizer			W/C	Water	Cement	Aggregate	Unit Weight (kg/m^3^)
	Type	%	kg					
X/1-0.65	Superplasticizer X	1	7.3	0.65	477	733	735	1952
X/1-0.70		1	7.3	0.7	496	708	710	1921
X/0.5-0.65		0.5	3.6	0.65	477	733	735	1949
X/0.5-0.70		0.5	3.6	0.7	496	708	710	1918
X/0.25-0.65		0.25	1.8	0.65	477	733	735	1947
X/0.25-0.70		0.25	1.8	0.7	496	708	710	1916
Y/1-0.65	Superplasticizer Y	1	7	0.65	477	733	735	1952
Y/1-0.70		1	7	0.7	496	708	710	1921
Y/0.5-0.65		0.5	3.5	0.65	477	733	735	1949
Y/0.5-0.70		0.5	3.5	0.7	496	708	710	1918
Y/0.25-0.65		0.25	1.75	0.65	477	733	735	1947
Y/0.25-0.70		0.25	1.75	0.7	496	708	710	1916

**Table 5:** Physical properties of limestone.

Property	Specification	Corse Aggregate	Fine Aggregate	Specification Limits
Specific gravity	ASTM C127 [19]	2.65	2.70	-
Water absorption, %	TS EN 1097/6 [20}	0.517	1.64	≤2.5
Soundness of aggregate by use of magnesium sulfate (%)	EN 1367-2 [21]	1.03	-	≤18
Flakiness index, %	BS 812-112 [22]	10.9	-	≤30
Los Angeles abrasion %	AASHTO T96 [23]	20.72	-	≤30
Methylene blue, g/kg	EN-933-9 [24]	-	1.5	≤2.0
Plasticity index	TS 1900-1 [25]	-	Non-Plastic	Non-Plastic
Organic substances, %	EN 1744-1 [26]	-	Negative	Negative

**Table 6:** Bitumen properties and specification limits.

Test	Specification	Results	Specification Limits
		50/70	50/70
Penetration (25 °C; 0.1 mm)	TS EN 1426 [27]	59	50–70
Softening point (°C)	TS EN 1427 [28]	52 °C	46–54
Penetration index (PI)	-	−0.54	-
Ductility (25 °C; 5 cm/min)	TS 119 [29]	>100	100
Flash point, °C (Cleveland open cup)	ISO 2592 [30]	280 °C	230 (min)
Thin Film Oven Test (TFOT) (163 °C; 5 h)	EN 12607-2 [31]	0.29	0.5 (max)
Change in softening point (°C)	TS EN 1427	3.1	9 (max)
Retained penetration (%)	TS EN 1426	60	50 (min)

**Table 7:** Calculating of blending formula for porous asphalt aggregates.

Sieve Size (mm)	%35A + %16B + %33C + %4D + %5E + %7F	Optimum Blend Passing (%)
19	100	100
12.5	0.35(3) + 0.16(98.7) + 33 + 4 + 5 + 7	65
9.5	0.35(0.6) + 0.16(11.7) + 0.33(99.5) + 4 + 5 + 7	51
4.75	0.34(0.6) + 0.16(0.6) + 0.33(11.2) + 0.04(99.9) + 5 + 7	19
2.36	0.35(0.6) + 0.16(0.6) + 0.33(1) + 0.04(17.3) + 0.05(98.8) + 7	13
1.18	0.35(0.6) + 0.16(0.6) + 0.33(0.9) + 0.04(1.9) + 0.05(13.5) + 7	8
0.6	0.35(0.6) + 0.16(0.6) + 0.33(0.9) + 0.04(1.7) + 0.05(2.7) + 0.07(99.2)	7
0.3	0.33(0.6) + 0.16(0.6) + 0.33(0.9) + 0.04(1.7) + 0.05(2.5) + 0.07(17.4)	2
0.15	0.35(0.5) + 0.16(0.6) + 0.33(0.9) + 0.04(1.7) + 0.05(2.5) + 0.07(4.7)	1.1
0.075	0.35(0.5) + 0.16(0.5) + 0.33(0.8) + 0.04(1.6) + 0.05(2.4) + 0.07(4.2)	1

**Table 8:** Physical properties of cellulosic fiber.

Compound	Compressed Cellulosic Fiber as Granular
Appearance	Granular fiber
Color	Brown
Pellet diameter	7 mm
Density	450 g/L
Average length	2 mm
Thermal resistance	˃250 °C

## Data Availability

The original contributions presented in this study are included in the article. Further inquiries can be directed to the corresponding author(s).
